# Highly Loaded and Binder-Free Molybdenum Trioxide Cathode Material Prepared Using Multi-Arc Ion Plating for Aqueous Zinc Ion Batteries

**DOI:** 10.3390/ma15175954

**Published:** 2022-08-29

**Authors:** Sainan Liu, Yangyang Sun, Jing Yang, Yi Zhang, Zhenyang Cai

**Affiliations:** 1 School of Minerals Processing and Bioengineering, Central South University, Changsha 410083, China; 2 School of Materials Science and Engineering, Central South University, Changsha 410083, China

**Keywords:** α-MoO_3_@CFC, binder-free, highly loaded, multi-arc ion plating, aqueous zinc-ion battery

## Abstract

Aqueous zinc-ion batteries (ZIBS) are becoming more popular as the use of energy storage devices grows, owing to advantages such as safety and an abundant zinc supply. In this study, molybdenum powder was loaded directly on carbon fiber cloth (CFC) via multi-arc ion plating to obtain Mo@CFC, which was then oxidatively heated in a muffle furnace for 20 min at 600 °C to produce high mass loading α-MoO_3_@CFC (α-MoO_3_ of 12–15 mg cm^−2^). The cells were assembled with α-MoO_3_@CFC as the cathode and showed an outstanding Zn^2+^ storage capacity of 200.8 mAh g^−1^ at 200 mA g^−1^ current density. The capacity retention rate was 92.4 % after 100 cycles, along with an excellent cycling performance of 109.8 mAh g^−1^ following 500 cycles at 1000 mA g^−1^ current density. Subsequently, it was shown that CFC-loaded α-MoO_3_ cathode material possessed significantly improved electrochemical performance when compared to a cell constructed from commercial MoO_3_ using conventional slurry-based electrode methods. This work presents a novel yet simple method for preparing highly loaded and binder-free cathodic materials for aqueous ZIBs. The results suggest that the highly loaded cathode material with a high charge density may be potentially employed for future flexible device assembly and applications.

## 1. Introduction

Lithium-ion batteries have been widely used in consumer gadgets and automobiles as a result of their excellent energy density. However, the inherent disadvantages, including lack of safety, increased cost, and environmental concerns have limited their applications in grid-scale energy storage [1,2,3]. Aqueous zinc-ion batteries (ZIBs) are seen as a viable alternative to lithium-ion batteries because of their benefits, such as a high theoretical capacity (820 mAh g^−1^), cost efficiency, environmental friendliness, and safety [4,5]. To date, various cathode materials, including manganese-based [6,7,8], vanadium-based [9,10,11,12], and Prussian blue analogues [13,14,15] have been investigated and used in aqueous ZIBs. During the preparation of electrode materials at the laboratory level, for a high mass specific capacity, an active material mass (such as mentioned above) of no more than 3 mg cm^−2^ may be commonly employed. It is still difficult to meet the demand for higher mass loading to achieve superior energy density and high capacity [16,17]. That is to say, further increasing the loading of active materials remains a great challenge. In addition, with the rapid development of flexible wearable devices, the research on flexible electrodes has attracted extensive attention [18,19,20]. Combining the two, a more suitable preparation of high-mass-loading flexible self-supporting electrode materials is needed, which not only can improve the capacity of aqueous ZIBs, but additionally promote the practical application of flexible wearable devices.

In this regard, several studies have used the in situ growth of active materials on flexible substrates to create cathode materials for ZIBs. For example, a series of manganese-based and vanadium-based cathode materials with a high mass loading using different synthetic methods (e.g., 3D printing and the hydrothermal method) have been studied [21,22,23,24]. Among various candidate cathode materials, orthogonal molybdenum trioxide (α-MoO_3_) is theoretically promising as a high mass loading electrode material for high-performance ZIBs because of its unique layered structure and remarkable thermodynamic stability [25,26]. To meet the requirements of high mass loading, multi-arc ion plating may be a feasible method, where the metal is directly evaporated on the solid cathode target by arc discharge, so as to deposit a thin film on the surface of the substrate [27,28]. To meet the requirements of flexibility, it is feasible to use carbon fiber cloth (CFC) as the deposition substrate [29,30]. In the current study, a novel method for producing an α-MoO_3_@CFC cathode with high mass loading and no binder via multi-arc ion plating and simple heat treatment is presented. This approach allows for a significant quantity of active material (about 12–15 mg cm^−2^) with a high binding strength to be placed on the CFC without the use of a conductive agent or binder, thereby improving the problems of active material shedding and the deterioration of battery performance. As a result, the prepared α-MoO_3_@CFC demonstrated an areal capacity of 2.61 mAh cm^−2^ at 200 mA g^−1^ current, and a higher energy density of 200.8 Wh kg^−1^ (a cathode energy density normalized to the cathode mass). This work may not only increase the ZIB capacity, but also facilitate the practical use of flexible wearable devices.

## 2. Experiments

### 2.1. Material Synthesis

A two-step procedure was used to fabricate the α-MoO_3_@CFC. First, the molybdenum target (Juno Metal Materials Co., Baoji, China) was placed in the multi-arc ion coating machine (SH007, Changsha Divine Arc Ion Coating Co., Changsha, China) along with the CFC, both of which had been sonicated with deionized water, acetone, and anhydrous ethanol. Following that, electrodeposition was initiated for 3 h, and the Mo@CFC was removed when the furnace temperature decreased to room temperature. The mass loading of Mo@CFC was about 8–10 mg cm^−2^. The mass ratio of Mo and CFC (about 11–13 mg cm^−2^) was about 0.62–0.91. Finally, Mo@CFC was oxidatively heated for 20 min in a muffle furnace at 600 °C to produce α-MoO_3_@CFC.

### 2.2. Material Characterization

Characterization of the specimens was conducted using an X-ray diffractometer (XRD, Rigaku DX-2500) and Cu Kα rays (λ = 1.54178Å) in the 3° to 80° range, and the morphology and structure were examined in detail using a scanning electron microscope (Nova Nano SEM 230) and a high-resolution transmission electron microscope (HRTEM, FEI Tecnai G2 F20, 200 kv).

### 2.3. Electrochemical Measurements

The batteries utilized in this work for testing electrochemical performance were all coin cells, type CR2016, and all of the constructed ones were run in air. The anode was made from a zinc foil with a 12 mm diameter, whereas the manufactured α-MoO_3_@CFC was cut into small square pieces of 1 cm × 1 cm for the cathode, with active material mass loading of about 12–15 mg cm^−2^. A glass microfiber filter (GFF Whatman) was employed as a separator, and the electrolyte was a 2 M ZnSO_4_ solution. They were then assembled layer by layer and sealed with a fully automated sealer (MSK-110D) before being kept at room temperature for 24 h for electrochemical testing.

The cathode for the commercial MoO_3_ cells was fabricated by coating a slurry of commercial MoO_3_ powder (purchased from Aladdin Industrial Corporation), polyvinylidene difluoride (PVDF), and acetylene black, with an 8:1:1 mass ratio on the stainless-steel mesh, which was cut in a 12 mm diameter. The cathode, electrolyte, separator, and assembly procedures are the same as described above. Moreover, for the battery testing equipment, galvanostatic charge–discharge measurements (LAND CT2001A) were made between 0.4 and 1.4 V. Cyclic voltammetry (CV) was carried out at a scan rate of 1 mV s^−1^ using an electrochemical workstation (CHI-600C, Shanghai, China). To conduct electrochemical impedance spectroscopy (EIS), a ZAHNER-IM6ex electrochemical workstation (ZAHNER Co., Kronach, Germany) was employed.

### 2.4. Results and Discussion

**Figure 1:**
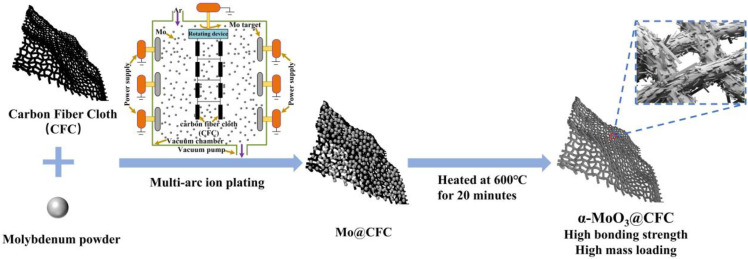
Schematic representation showing the synthesis of α-MoO_3_@CFC.

Figure 1 schematically represents a basic two-step synthesis of α-MoO_3_@CFC. Mo@CFC is directly produced by multi-arc ion plating. As a molybdenum source, six pure molybdenum targets (99.99% purity) are evenly distributed on both sides of the vacuum chamber, whereas the treated CFC is fixed as a carrier on a rotating rack located at the upper end of the vacuum chamber, allowing molybdenum to be uniformly plated on. The Mo@CFC is then placed within a quartz crucible and heated for 20 min at 600 °C, allowing Mo@CFC to fully oxidize to α-MoO_3_@CFC.

As depicted by the Mo@CFC and α-MoO_3_@CFC in Figure S1 of the Supporting Information, a layer of off-white α-MoO_3_ evenly covers the surface of the black pure CFC following 20 min of heating at 600 °C, forming α-MoO_3_@CFC with good flexibility.

**Figure 2:**
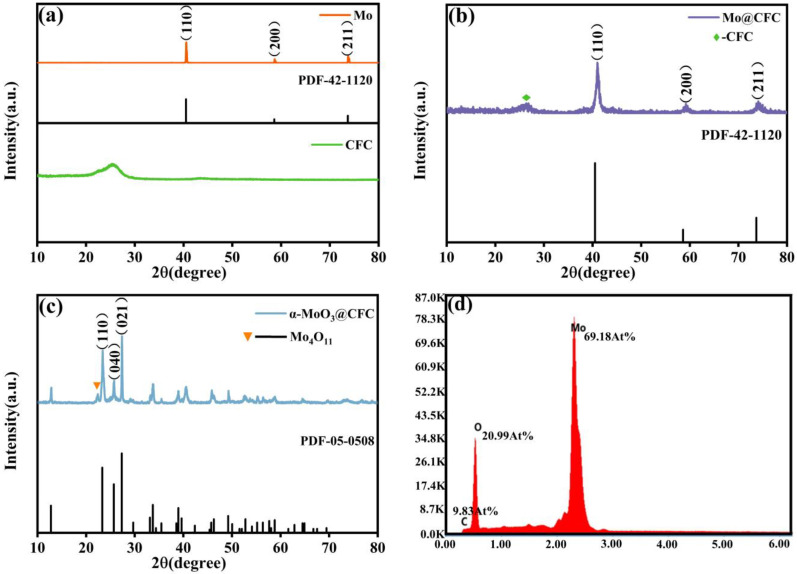
The patterns of X-ray diffraction for (**a**) Mo powder and pure CFC, and (**b**) Mo@CFC; (**c**,**d**) X-ray diffraction and EDS of α-MoO_3_@CFC.

All of the Mo@CFC diffraction peaks are in line with the standard PDF card 42-1120, as shown in Figure 2b. Figure 2a,b suggest that not only Mo is uniformly plated on the surface of CFC, but the native structures of Mo and CFC are not disrupted throughout the preparation process. All of the diffraction peaks of MoO_3_ are indexed well to orthorhombic α-MoO_3_ (JCPDS No.05-0508), with high crystallinity. In contrast, there is a diffraction peak attributed to Mo_4_O_11_ (JCPDS No. 05-0337), indicative of the existence of low-valence-state Mo after the oxidation treatment. The EDS result (Figure 2d) also suggests that a high purity α-MoO_3_@CFC was prepared.

The XRD patterns of α-MoO_3_@CFC at various heating temperatures are presented in Figure S2a. When the temperature increases, the crystallinity of α-MoO_3_ increases gradually. At 600°C, the characteristic peak of Mo disappears, which indicates that Mo loaded on CFC is completely oxidized to α-MoO_3_ at this temperature. Figure S2b displays the XRD patterns of α-MoO_3_@CFC heated for different times at 600 °C (40 min and 60 min). With the increase in heating time, the oxidation of Mo is more complete. At 60 min, the characteristic peak of Mo_4_O_11_ disappears. However, with the extension of time, the CFC was destroyed by oxidation, and the mechanical properties of the samples weakened. Therefore, 20 min was determined as being the optimal preparation condition.

**Figure 3:**
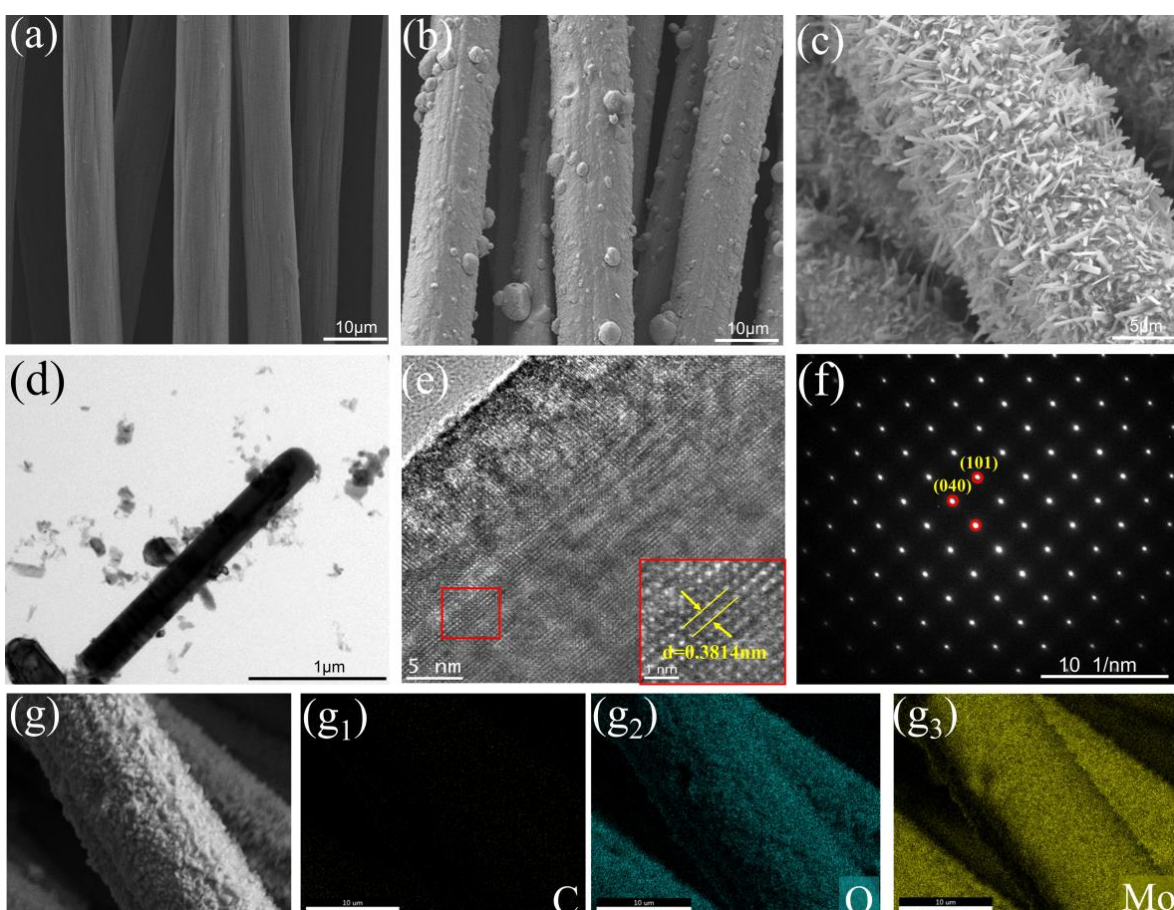
SEM image of (**a**) CFC, (**b**) Mo@CFC, and (**c**) α-MoO_3_@CFC heated at 600 °C for 20 min; (**d**–**g**) TEM image, HRTEM image, SAED image, and EDS mapping of C (**g1**), O (**g2**), and Mo (**g3**) of the α-MoO_3_@CFC.

SEM images of pure CFC, Mo@CFC, and α-MoO_3_@CFC are shown in Figure 3a–c. Because of the enormous amount of Mo powder present on the surface, the diameter of the previously smooth CFC becomes much thicker following multi-arc ion plating. After oxidation, α-MoO_3_@CFC with a one-dimensional rod-like shape are obtained, as presented in Figure 3c. Furthermore, the SEM image of commercial MoO_3_ is shown in Figure S3, presenting an irregular morphology. The TEM image further confirms the typical rod-like structure, with a uniform width of about 25 nm and a length of up to about 2 μm. As presented in Figure 3e, the HRTEM image displays clear lattice fringes with a d-spacing of 0.3814 nm, in good agreement with the (200) crystalline planes of the orthorhombic MoO_3_. Moreover, the rod-like structure demonstrates the monocrystalline structure, as indicated by the SAED pattern (Figure 3f). The energy dispersive spectroscopy (EDS) elemental mapping images show the compositions of Mo, O, and C (Figure 3g,g1–g3). It is worth mentioning that the distribution of C is relatively unapparent, due to the high loading on CFC.

**Figure 4:**
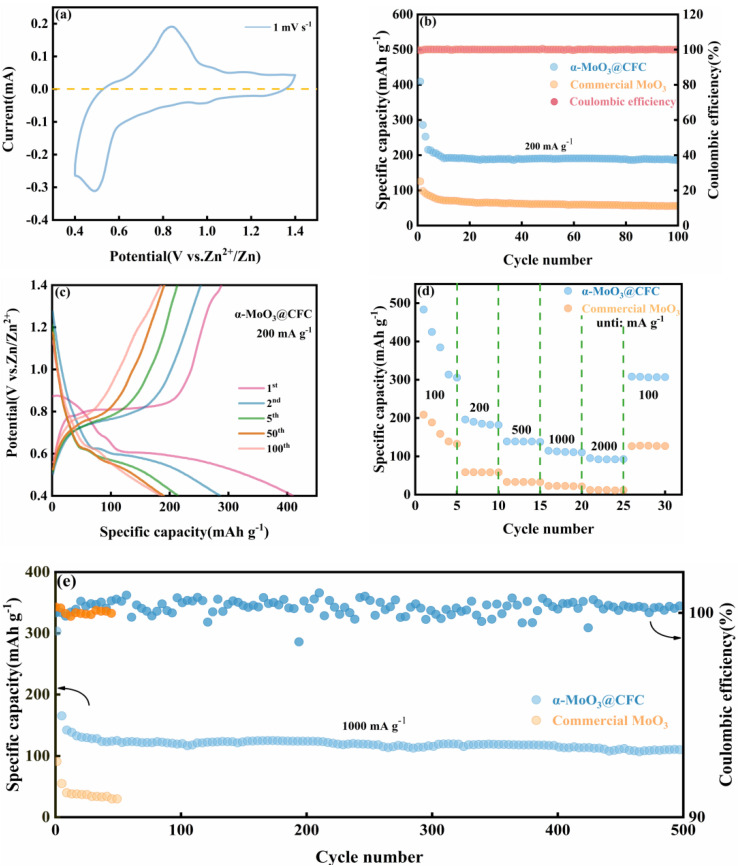
Electrochemical performance of α-MoO_3_@CFC and commercial MoO_3_. (**a**) Presented are 1 mV s^−1^ CV curves of α-MoO_3_@CFC, (**b**) cycle performance of α-MoO_3_@CFC and commercial MoO_3_ powder at 200 mA g^−^^1^ density, (**c**) galvanostatic charge–discharge curves of α-MoO_3_@CFC, (**d**) rate performance of α-MoO_3_@CFC and commercial MoO_3_ powders. (**e**) Long-term cycling performance of α-MoO_3_@CFC and commercial MoO_3_ powders at a current density of 1000 mA g^−1^.

The electrochemical performances of ZIBs containing α-MoO_3_@CFC and commercial MoO_3_ as active cathode materials are shown in Figure 4. Figure 4a and Figure S5 display the classic CV curves for α-MoO_3_@CFC and commercial MoO_3_ in the potential window of 0.4–1.4 V, at a 1 mV s^−1^ scan rate. Figure 4a represents a group of redox peaks located at around 0.83 V and 0.48 V, indicating typical battery behavior, with the oxidation peak situated around 0.83 V arising from the extraction of Zn^2+^ from α-MoO_3_@CFC; and the reduction peak located near 0.48 V can be assigned to the insertion of Zn^2+^ into MoO_3_ [25]. A comparison of the cycling performance of α-MoO_3_@CFC and commercial MoO_3_ powder at a current density of 200 mA g^−1^ is shown in Figure 4b. The initial commercial MoO_3_ has a specific capacity of just 125.6 mAh g^−1^ and decays to 55.8 mAh g^−1^ following 100 cycles, with a capacity retention rate amounting to 44.4%. In contrast, the specific capacity of α-MoO_3_@CFC stabilizes at around 200 mAh g^−1^ after 100 cycles, with a capacity retention rate of 92.4%. Additionally, the calculated areal capacity of this Zn//α-MoO_3_@CFC battery reaches 2.61 mAh cm^−2^ (200.8 mAh g^−1^ based on the average mass of 13 mg cm^−2^), with an excellent energy density of 200.8 Wh kg^−1^.

Figure 4c shows the curves of the α-MoO_3_@CFC-containing cathode at the 1st, 2nd, 5th, 50th, and 100th cycles, charged and discharged at 200 mA g^−1^. The initial discharge and charge capacities were equal to 409.3 and 288 mA h g^−1^, respectively. These values correspond to a Coulombic efficiency of 70.4%. The series of charge/discharge plateaus agree well with the CV curves shown in Figure 4a. More notably, the rate performance of α-MoO_3_@CFC is significantly improved in comparison to that of commercial MoO_3_, as is evident in Figure 4d, with specific capacities of 483.4, 196.2, 138.8, 114.6, and 95.4 mAh g^−1^ at current densities ranging from 100 mA g^−1^ to 2000 mA g^−1^, respectively. The specific capacity increased to 308.2 mAh g^−1^ when the current density was returned to 100 mA g^−1^, and it remained steady in future cycles. The long-term cycling performance of α-MoO_3_@CFC at a current density of 1000 mA g^−1^ is shown in Figure 4e, where the specific capacity of α-MoO_3_@CFC remained at 109.8 mAh g^−1^ following 500 cycles, whereas the commercial MoO_3_ had poor performance, with a rapid decline of the capacity. In addition, the ex situ SEM images of α-MoO_3_@CFC and commercial MoO_3_ after 20 cycles at a current density of 1000 mA g^−1^ are shown in Figure S4. Obviously, the mechanical stability of α-MoO_3_@CFC is better.

**Figure 5:**
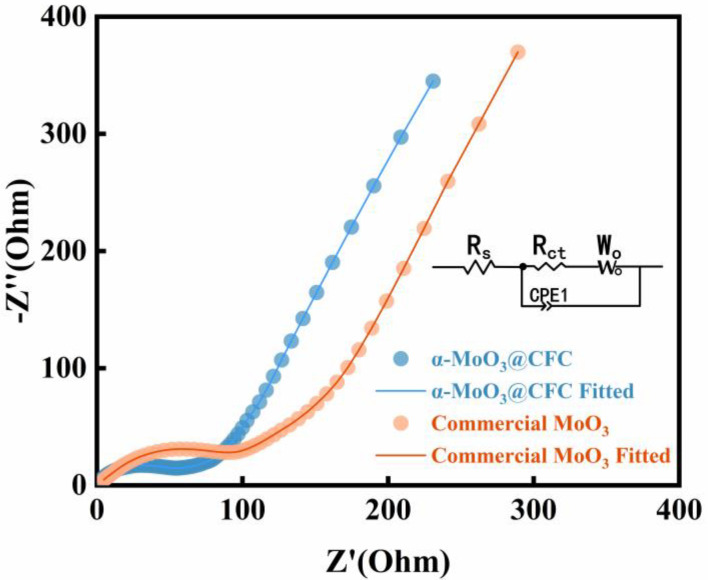
Electrochemical impedance spectroscopy of α-MoO_3_@CFC and commercial MoO_3_.

In order to better comprehend the excellent electrochemical performance of the α-MoO_3_@CFC electrode, electrochemical impedance spectroscopy (EIS) measurements are shown in Figure 5. The Nyquist plots of both α-MoO_3_@CFC and commercial MoO_3_ consist of semicircles at high frequencies and a straight line at lower frequencies. These regions correspond to the resistance of the electrode surface (R_f_) and a charge-transfer resistance (R_ct_), respectively [26,31,32]. Fitting the EIS data obtained for the α-MoO_3_@CFC and commercial MoO_3_ electrodes before cycling showed that R_ct_ values of the cathodes based on the α-MoO_3_@CFC and commercial MoO_3_ materials are equal to 43.73 and 82.09 Ω, respectively. These results confirmed that α-MoO_3_@CFC possessed better conductivity, as well as stronger ion and electron migration ability, than commercial MoO_3_. From a comparison of this work with some reported studies (high mass loading), in some respects, α-MoO_3_@CFC-based electrodes for ZIBs have possible application prospects (see Table 1). The main reason for the improved performance may be the strategy of a binder-free electrode.

## 3. Conclusions

In summary, ZIBs are fabricated with a high mass loading (12–15 mg cm^−2^) and binder-free cathode material, α-MoO_3_@CFC, prepared using multi-arc ion plating and simple heating. When operated at a current density of 200 mA g^−1^, the α-MoO_3_@CFC electrode has an electrochemical specific capacity of 200.8 mAh g^−1^ after 100 cycles. After 500 cycles at a current density of 1000 mA g^−1^, it still has a capacity of 109.8 mAh g^−1^. The exceptional mechanical characteristics and high areal capacity of 2.61 mAh cm^−2^ with an excellent energy density of 200.8 Wh kg^−1^ reveal considerable promise for flexible wearable and grid energy storage applications.

## Figures and Tables

**Table 1:** Comparison of electrochemical properties of α-MoO_3_@CFC and reported aqueous zinc-ion cathode materials.

Type of Material	Cycle Number, Cycling Capacity (mAh g^−1^) (Current Density (mA g^−1^))	Mass Loading (mg cm^−2^)	Ref.
α-MoO_3_@CFC	100th, 200.8 (200) 500th, 109.8 (1000)	12–15	This Work
MoO_3_ nanowires	400th, 171.14 (400)	11.1	[25]
MoO_3_ nanobelt	100th, 254.77 (100)	4.4–4.5	[33]
V_3_O_7_⋅H_2_O nanoarray	50th, 323 (100) 800th, 155 (2000)	5	[21]
H_11_Al_2_V_6_O_23.2_@graphene	400th, 131.7 (2000)	15.7	[24]
3D printed FeVO/rHGO	650th, 126.4 (2000)	12.4	[23]
MnO_2_ composite electrode	300th, 184 (200)	9.5	[16]

## Data Availability

The data presented in this study are available on reasonable request from the corresponding author.

## Supplementary Materials

The following supporting information can be downloaded at: https://www.mdpi.com/article/10.3390/ma15175954/s1, Figure S1: Macroscopic morphology of (a) Mo@CFC, (b) α-MoO_3_@CFC. Figure S2: XRD patterns of α-MoO_3_@CFC at (a) different heating temperatures, (b) different heating times at 600°C. Figure S3: SEM image of commercial MoO_3_. Figure S4: The ex-situ SEM images of α-MoO_3_@CFC (a) and commercial MoO_3_ (b) after 20 cycles at a current density of 1000 mA g^−1^. Figure S5: 1 mV s^−1^ CV curves of commercial MoO_3_.
