# Supplementary material for: Highly Loaded and Binder-Free Molybdenum Trioxide Cathode Material Prepared Using Multi-Arc Ion Plating for Aqueous Zinc Ion Batteries
Source: Materials (Basel). 2022 Aug 29;15(17):5954. doi: 10.3390/ma15175954 (PMC9457491; doi:10.3390/ma15175954)
Supplement: Supplementary file 1 [file materials-15-05954-s001.zip › materials-1865800-Supplementary.pdf]

Supporting information

Highly Loaded and Binder-Free Molybdenum Trioxide Cathode Material Prepared Using Multi-Arc Ion Plating for Aqueous Zinc Ion Batteries

Sainan Liu [1,*], Yangyang Sun [1], Jing Yang [1], Yi Zhang [1,*] and Zhenyang Cai [2,*]

[1] School of Minerals Processing and Bioengineering, Central South University, Changsha 410083, China
[2] School of Materials Science and Engineering, Central South University, Changsha 410083, China
* Correspondence: liusainanbaby@163.com (S.L.); yee_z10@csu.edu.cn (Y.Z.); csuczy@csu.edu.cn (Z.C.)

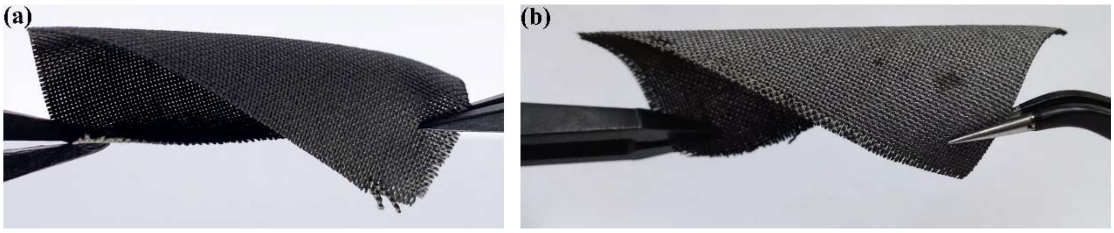

Figure S1. Macroscopic morphology of (a) Mo@CFC, (b) α-MoO_3@CFC.

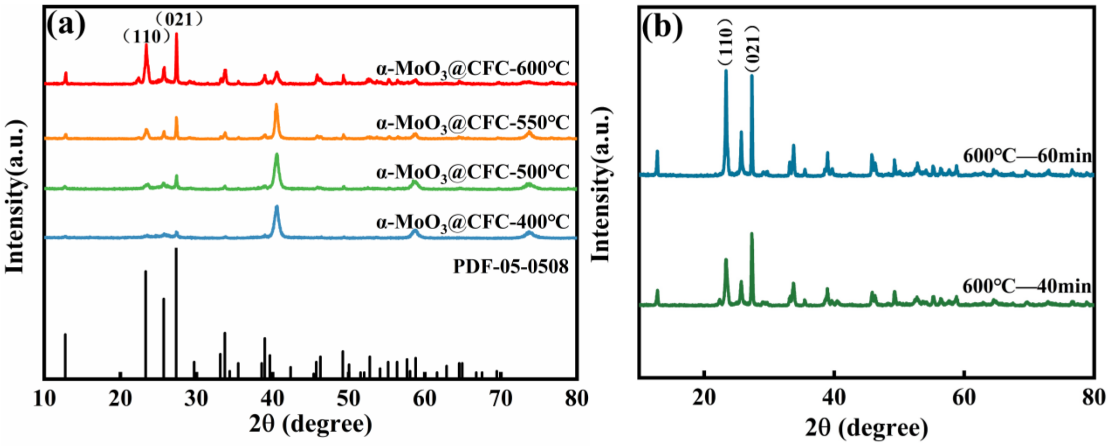

Figure S2. XRD patterns of α-MoO_3@CFC at (a) different heating temperatures, (b) different heating times at 600°C.

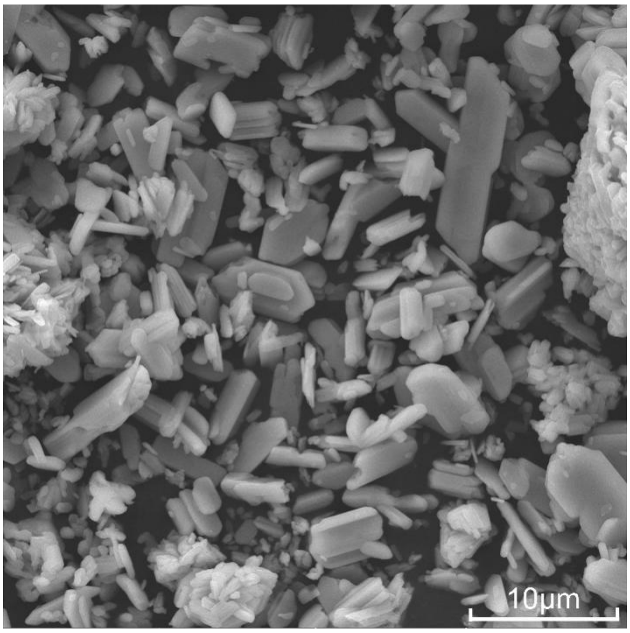

Figure S3. SEM image of commercial MoO_3.

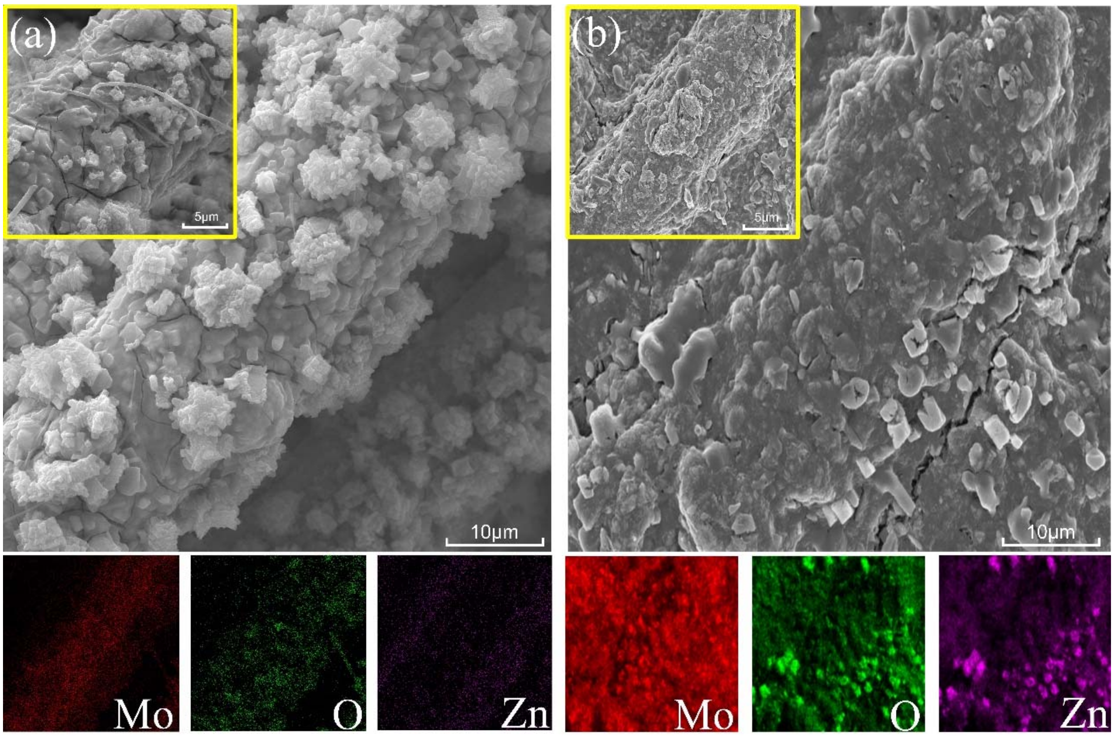

Figure S4. The ex-situ SEM images of α-MoO_3@CFC(a) and commercial MoO_3(b) after 20 cycles at a current density of 1 A g^{-1}.

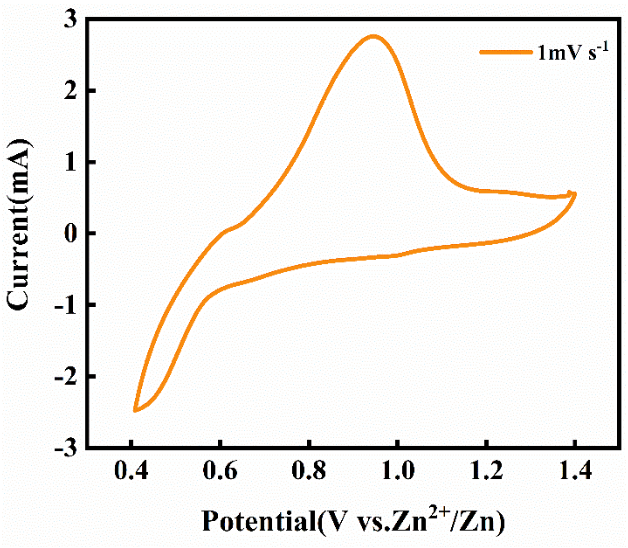

Figure S5. 1 mV s^{-1} CV curves of commercial MoO_3.
